# Smoking history and its relationship with comorbidities in patients with obstructive sleep apnea

**DOI:** 10.18332/tid/123429

**Published:** 2020-07-01

**Authors:** Chuan Shao, Huan Qi, Qing Fang, Jinjing Tu, Qianjun Li, Ling Wang

**Affiliations:** 1 Department of Pulmonary and Critical Care Medicine, Ningbo Medical Center Lihuili Hospital, Ningbo, China; 2 Department of Special Procurement Ward, The First Affiliated Hospital of Soochow University, Suzhou, China; 3 Department of General Practice, The First Affiliated Hospital of Soochow University, Suzhou, China; 4 Sleep Medicine Center, Nanfang Hospital, Southern Medical University, Guangzhou, China; 5 Department of Pulmonary and Critical Care Medicine, Ningbo Yinzhou No. 2 Hospital, Ningbo, China

**Keywords:** obstructive sleep apnea, smoking, comorbidities, polysomnography

## Abstract

**INTRODUCTION:**

Current knowledge on the correlation between smoking and comorbidities associated with obstructive sleep apnea (OSA) is limited. This study evaluated the smoking history of OSA patients and analyzed the association between smoking and OSA comorbidities.

**METHODS:**

Retrospective analysis was performed in newly diagnosed OSA patients in our hospital, a tertiary medical center, from January 2016 to December 2019. In all, 1021 patients were enrolled and divided into two groups, non-smokers (n=796) and current/former smokers (n=225), in order to compare their clinical manifestations and polysomnographic results and to analyze the association between smoking and comorbidities.

**RESULTS:**

Compared with the non-smokers, the current/former smokers had higher Epworth sleepiness scale (ESS) scores (9.3 ± 4.0 vs 8.5 ± 5.1; p<0.05), longer sleep latency (SL) [20.5 (12.3–39.3) vs 18.5 (10.0–34.0) minutes; p<0.05], and a lower nocturnal mean oxygen saturation (91.8 ± 3.6% vs 92.8 ± 3.4%; p<0.001). There was no significant difference in the apnea-hypopnea index (AHI) between the two groups. OSA patients with a history of smoking had significantly increased risk of hypertension (OR=2.09; 95% CI: 1.46– 3.01), chronic obstructive pulmonary disease (COPD) (OR=9.80; 95% CI: 4.73–20.33), gastroesophageal reflux disease (GERD) (OR=1.97; 95% CI: 1.19–3.27), and chronic pharyngitis (OR=1.83; 95% CI: 1.32–2.54).

**CONCLUSIONS:**

No significant association was found between previous smoking history and current OSA severity. OSA patients with a history of smoking had an increased risk of hypertension, COPD, GERD, and chronic pharyngitis.

## INTRODUCTION

Obstructive sleep apnea (OSA) is the most common breathing disorder during sleep, characterized by recurrent upper airway obstruction/collapse, chronic intermittent hypoxia, and fragmented sleep. With the increase in obesity and aging within the global population, the prevalence of OSA has increased dramatically^1^. A recent study has estimated that approximately 936 million adults worldwide aged 30–69 years suffer from OSA, and China has the highest number of OSA patients in the world^2^. It is estimated that approximately 176 million Chinese adults aged 30–69 years have OSA, and the number of moderate to severe OSA patients has reached 66 million^2^. OSA triggers damage in multiple organs/systems through a variety of pathophysiological mechanisms, such as chronic intermittent hypoxia (CIH), fluctuation in intrathoracic pressure, sympathetic activation, oxidative stress and inflammation, and endothelial dysfunction^1^. Untreated OSA is associated with a variety of adverse clinical consequences and outcomes, such as cardiovascular diseases, metabolic disorders, neuropsychiatric disorders, gastroesophageal reflux disease (GERD), and malignant tumor^3,4^.

The relation between OSA and smoking is inconclusive, and results from previous studies are inconsistent. One study showed that compared with non-smoking OSA patients, smoking OSA patients had a higher apnea-hypopnea index (AHI), a lower nocturnal mean oxygen saturation, and a higher Epworth Sleepiness Scale (ESS) score, suggesting that smoking was related to the severity of OSA^5^. A previous study has suggested that the AHI increases with the increase in smoking rate^6^. OSA patients who formerly smoked had longer period of hypoxia during sleep than that of non-smoking OSA patients. This phenomenon was more severe in OSA patients who were heavy smokers. In contrast, Hsu et al.^7^ and Casasola et al.^8^ showed that smoking had no correlation with OSA. A recent meta-analysis of a total of 5264 participants in 14 studies suggested that smoking was not related to OSA, but the level of evidence in the included studies was low^9^.

Smoking is also one of the important risk factors for many complications of OSA, such as cardiovascular and cerebrovascular diseases, metabolic disorders, and GERD^10-12^. Few studies have investigated the pathophysiological changes caused by the overlap of OSA and smoking. There are few clinical studies on the relationship between smoking status and comorbidities in OSA patients. Studies by Zhu et al.^13^ have shown that smoking and OSA jointly cause insulin resistance and have synergistic effects on metabolic disorders. Lui et al.^14^ have also suggested that the coexistence of CIH and smoking causes severe endothelial dysfunction. Studies have also suggested that coexistence of both OSA and smoking leads to more severe cognitive dysfunction than any single factor^15^. However, Bielicki et al.^5^ did not show that smoking OSA patients had an increased proportion of type 2 diabetes, coronary artery disease (CAD), myocardial infarction, and chronic heart failure. Instead, their study showed that non-smoking patients had a higher incidence of hypertension.

Most previous studies were conducted in developed western countries, and the tested subjects were mostly Caucasian and black populations. Very few studies have been conducted in Asia, with the tested subjects being Asian populations or specifically Chinese patients. Chinese OSA patients have their particularities. For example, compared with their counterparts in western countries, Chinese OSA patients have a lower body mass index (BMI), which we have shown in two previous studies^16,17^. This not only represents the characteristics of Chinese patients, but also to some extent, the characteristics of relatively lean OSA patients in East Asia. This is the first study from China to explore the relationship between smoking and comorbidities associated with OSA. This study aims to: 1) assess the previous smoking history of newly diagnosed OSA patients; 2) compare the clinical manifestation and polysomnographic results of OSA patients with and without smoking history; and 3) analyze the relationship between smoking and comorbidities associated with OSA.

## METHODS

### Study design

This was a single-center retrospective observational study that included newly diagnosed OSA patients in a tertiary medical center in China from January 2016 to December 2019. The detailed information of patients, such as demographic characteristics, smoking history, and OSA-related symptoms, signs, peripheral capillary oxygen saturation (SpO_2_) when awake before sleep, and comorbidities, were extracted from the hospital information system (HIS) and the main polysomnographic results of the patients were collected. The diagnosis of each comorbidity was based on the corresponding diagnostic criteria or a clear history of previous diagnoses and treatments. Since the detailed cigarette pack-year history was not obtained at the time of patients’ visit, only the ever-smoking or never-smoking status was documented. We combined current and former smokers into one group, and non-smokers into another. The clinical manifestations and polysomnographic results of the two groups were compared and the relationship between smoking history and comorbidities associated with OSA was analyzed. This study was approved by the ethics committee of Ningbo Medical Center Lihuili Hospital (ethical approval No: KY2020PJ016). This is a retrospective study, and for this type of study formal consent is not required. Every patient managed in the sleep laboratory in our hospital was asked to give signed consent for the potential use of his/her clinical data for research purposes. The raw data of this study are available from the corresponding author, upon request.

### Study population

**Figure 1:**
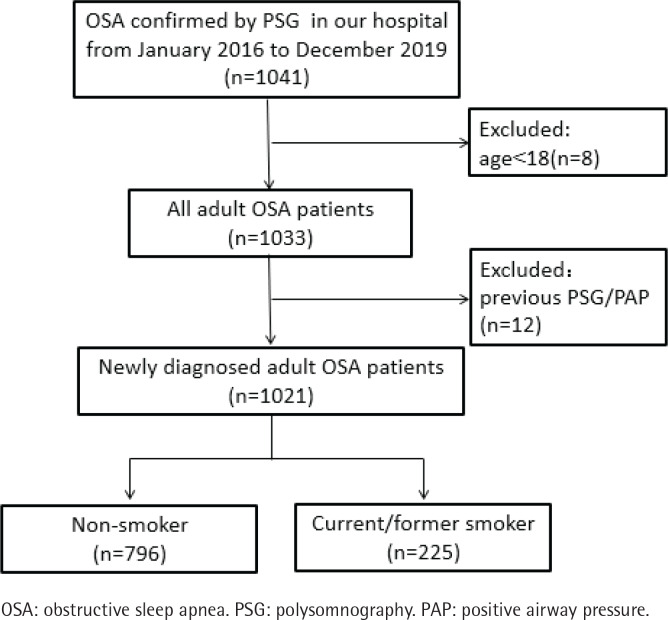
Flow chart of the participants of the study

Patients with newly diagnosed OSA in our hospital from January 2016 to December 2019 were included in this study. The exclusion criteria of this study were: 1) patients aged <18 years; and 2) patients had previously received a snoring or OSA-related diagnosis and treatment. This study included a total of 1021 patients: 863 males and 158 females; 64 elderly cases (≥65 years) and 957 non-elderly cases; 796 non-smokers and 225 current/former smokers (Figure 1, Table 1).

**Table 1:** Sociodemographic characteristics of OSA patients with different smoking histories

*Characteristics*	*Non-smoker (N=796)*	*Current/former smoker (N=225)*	*t or χ2 test*	*p*
**Male patients,** n (%)	650 (81.7)	213 (94.7)	22.693	<0.001*
**Elderly patients,** n (%)	40 (5)	24 (10.7)	9.502	0.002*
**Age** (years), mean ± SD	45.0 ± 11.3	46.7 ± 12.3	−1.994	0.046*
B**M**I (kg/m^2^), mean ± SD	27.2 ± 3.4	27.1 ± 3.2	0.562	0.574
**SpO**_2_ **when awake** (%), mean ± SD	96.7 ± 1.0	96.7 ± 1.0	−0.876	0.381
**ESS score,** mean ± SD	8.5 ± 5.1	9.3 ± 4.0	−2.293	0.022*

OSA: obstructive sleep apnea. BMI: body mass index. SpO_2_: peripheral oxygen saturation. ESS: Epworth sleepiness score.

* p-values indicate statistical significance.

### OSA diagnosis

We used an ESS score to assess daytime sleepiness^18^. The ESS score was completed by a sleep technician on the night of the polysomnography (PSG). All patients received an overnight PSG (Alice 5, Respironics, Philips, Pittsburgh, PA). Apnea is defined as the amplitude of oronasal thermal airflow that is reduced by more than 90% from baseline for at least 10 seconds. Hypopnea is defined as the amplitude of oronasal pressure airflow that is reduced by more than 30% from baseline, with a decrease in SpO_2_ by 3% or arousal for at least 10 seconds. The interpretation of sleep stages, micro-arousals, and respiratory events were based on the AASM Manual for the Scoring of Sleep and Associated Events version 2.5^19^. OSA diagnosis was based on The Third Edition of International Classification of Sleep Disorders (ICSD-3) issued by the American Academy of Sleep Medicine (AASM)^20^ (i.e. AHI ≥5 with OSA-related symptoms; or AHI ≥15 with/without OSA-related symptoms). Main results of PSG were recorded, including sleep efficiency, wake after sleep onset (WASO), sleep latency (SL), the proportion of each sleep stage, micro-arousal index (MAI), AHI, maximum apnea duration, nadir SpO_2_, mean SpO_2_, oxygen desaturation index (ODI), and saturation impair time below 90% (SIT_90_).

### Statistical analysis

Normally distributed continuous data are presented as mean ± standard deviation while non-normally distributed data are presented as median and interquartile range. The categorical data are presented as the number of cases (and as percentages). The comparison between two groups of continuous and normally distributed data were performed by independent sample t-test. The comparison between two groups non-normally distributed data was performed by the Mann-Whitney test. The comparison between two groups of categorical variable data was performed using a chi-squared test. The power regarding the proportion of subjects with a comorbid disease, was calculated based on a two-sided chi-squared test. With a sample size of 796 non-smokers and 225 current/former smokers, the study had more than 90% power to detect a difference. Multivariate analysis was performed using logistic regression analysis to investigate the association between smoking history and comorbidities associated with OSA. The covariables included in the regression model for multivariate analysis are: gender, age, BMI, ESS score, smoking history, hypertension, CAD, chronic obstructive pulmonary disease (COPD), GERD, chronic pharyngitis, SL, MAI, AHI, nadir SpO_2_, mean SpO_2_, ODI, and SIT_90_. The comorbid diseases were not included as covariables when they were treated as dependent variables. SPSS 22.0 (IBM SPSS Inc., Chicago, IL, USA) was used for the statistical analysis. PASS 11.0 (NCSS, Kaysville, Utah, USA) was used for power analysis. A p<0.05 was considered statistically significant.

## RESULTS

### Sociodemographic characteristics

Of the 1021 OSA patients included in this study, there were 796 (78%) non-smokers and 225 (22%) current/former smokers, with males accounting for the vast majority in both groups. However, the proportion of men was higher in the current/former smoker group than in the non-smoker group (94.7% vs 81.7%). Compared to the non-smokers, the mean age of the current/former smokers was higher (46.7 ± 12.3 vs 45.0 ± 11.3 years), and the proportion of elderly patients was also higher (10.7% vs 5%). No significant differences in BMI and SpO_2_ when awake were found between the two groups. The ESS score in the current/former smokers was higher than that of the non-smokers (9.3 ± 4.0 vs 8.5 ± 5.1) (Table 1).

### Clinical manifestations and comorbidities

The current/former smokers had higher ratios of associated hypertension (45.3% vs 32.0%), CAD (13.3% vs 7.7%), COPD (15.1% vs 2.8%), GERD (15.6% vs 8.7%), and chronic pharyngitis (50.2% vs 41.0%) than non-smokers. OSA-related symptoms, such as nighttime awakenings, morning headaches, dry mouth, and some other comorbidities including type 2 diabetes mellitus (T2DM) and stroke were not significantly different between the two groups (Table 2).

**Table 2:** Clinical manifestations and comorbidities of OSA patients with different smoking histories

*Manifestations Comorbidities*	*Non-smoker (N=796) n (%)*	*Current/former smoker (N=225) n (%)*	*χ^2^ test*	*p*
Nighttime awakenings	305 (38.3)	86 (38.2)	0.001	0.979
Morning headaches	144 (18.1)	32 (14.2)	1.840	0.175
Dry mouth	512 (64.3)	144 (64.0)	0.008	0.929
Nocturia	223 (28.0)	66 (29.3)	0.150	0.698
Decline of memory	485 (60.9)	135 (60.0)	0.064	0.801
Irritability	281 (35.3)	79 (35.1)	0.003	0.985
Impact on life	310 (38.9)	92 (40.9)	0.278	0.598
Impact on career	225 (28.3)	53 (23.6)	1.965	0.161
Impact on transportation	125 (15.7)	29 (12.9)	1.085	0.298
Hypertension	255 (32.0)	102 (45.3)	13.642	<0.001*
T2DM	71 (8.9)	20 (8.9)	0.000	0.989
CAD	61 (7.7)	30 (13.3)	6.946	0.008*
COPD	22 (2.8)	34 (15.1)	51.588	<0.001*
Stroke	18 (2.3)	7 (3.1)	0.530	0.466
GERD	69 (8.7)	35 (15.6)	9.095	0.003*
Hypothyroidism	29 (3.6)	7 (3.1)	0.146	0.702
Chronic pharyngitis	326 (41.0)	113 (50.2)	6.147	0.013*
Rhinitis	260 (32.7)	83 (36.9)	1.404	0.236
Enlarged tonsil	63 (7.9)	24 (10.7)	1.704	0.192
Pharyngeal stenosis	192 (24.1)	57 (25.3)	0.140	0.708

OSA: obstructive sleep apnea. T2DM: type 2 diabetes mellitus. CAD: coronary artery disease. GERD: gastroesophageal reflux disease. COPD: chronic obstructive pulmonary disease.

* p-values indicate statistical significance.

SL of the current/former smokers was higher than in non-smokers [20.5 (12.3–39.3) vs 18.5 (10.0–34.0) min], while the nocturnal mean oxygen saturation was lower than in non-smokers (91.8 ± 3.6% vs 92.8 ± 3.4%). No significant differences in the AHI and other polysomnographic parameters were found between the two groups (Table 3).

**Table 3:** Polysomnographic results of OSA patients with different smoking histories

*Measurements*	*Non-smoker (N=796)*	*Current/former smoker (N=225)*	*t or Z*	*p*
Sleep efficiency (%)	85.6 ± 11.7	85.0 ± 12.2	0.627	0.531
WASO (min)	27.3 (13.0–62.9)	28.0 (13.0–72.8)	−0.525	0.599
SL (min)	18.5 (10.0–34.0)	20.5 (12.3–39.3)	−1.986	0.047*
Stage N1 ratio (%)	42.6 ± 18.1	42.1 ± 18.8	0.361	0.718
Stage N2 ratio (%)	43.8 ± 15.1	42.8 ± 15.6	0.954	0.340
Stage N3 ratio (%)	18.5 (10.0–34.0)	20.5 (12.3–39.3)	−1.689	0.091
Stage REM ratio (%)	7.3 (1.3–12.2)	7.0 (0.6–13.2)	−0.041	0.967
MAI (h^−1^)	43.4 ± 19.2	44.1 ± 22.0	−0.484	0.629
AHI (h^−1^)	43.6 ± 24.2	44.5 ± 26.8	−0.418	0.676
Maximum apnea duration (s)	54.9 ± 21.9	55.4 ± 25.0	−0.286	0.775
Nadir SpO_2_ (%)	71.7 ± 13.2	70.2 ± 14.8	1.383	0.168
Mean SpO_2_ (%)	92.8 ± 3.4	91.8 ± 3.6	3.835	<0.001*
ODI (h^−1^)	44.1 (22.8–64.7)	40.7 (23.6–63.9)	−0.186	0.853
SIT_90_ (%)	8.5 (1.6–23.7)	8.7 (1.5–20.9)	−0.092	0.926

Values are given as mean ± standard deviation or median and interquartile range. OSA: obstructive sleep apnea. WASO: wake after sleep onset. SL: sleep latency. REM: rapid eye movement. MAI: micro-arousal index. AHI: apnea hypopnea index. SpO_2_: peripheral oxygen saturation. ODI: oxygen desaturation index. SIT_90_: saturation impair time below 90%.

* p-values indicate statistical significance.

### Relationship between smoking history and comorbidities

Multivariate analysis showed that smoking history had the strongest association with COPD (OR=9.80; 95% CI: 4.73–20.33), followed by hypertension (OR=2.09; 95% CI: 1.46–3.01), GERD (OR=1.97; 95% CI: 1.19–3.27), and chronic pharyngitis (OR=1.83; 95% CI: 1.32–2.54) (Table 4). In univariate analysis, the proportion of associated CAD varied significantly in the two groups. However, smoking was not independently associated with CAD in multivariate analysis.

**Table 4:** Logistic regression analysis on the relationship between smoking history and comorbidities associated with OSA

*Comorbidities*	*Univariate*			*Multivariate*		

	*OR*	*95% CI*	*p*	*OR*	*95% CI*	*p*
Hypertension	1.76	1.30–2.38	<0.001*	2.09	1.46–3.01	<0.001*
T2DM	1.00	0.59–1.68	0.989	0.74	0.40–1.36	0.333
CAD	1.85	1.17–2.95	0.009*	1.28	0.74–2.23	0.380
COPD	6.26	3.56–10.96	<0.001*	9.80	4.73–20.33	<0.001*
Stroke	1.39	0.57–3.37	0.468	1.06	0.35–3.23	0.922
GERD	1.94	1.25–3.00	0.003*	1.97	1.19–3.27	0.008*
Hypothyroidism	0.849	0.37–1.97	0.703	0.96	0.37–2.53	0.940
Chronic pharyngitis	1.46	1.08–1.96	0.013*	1.83	1.32–2.54	<0.001*
Rhinitis	1.21	0.89–1.64	0.236	1.24	0.87–1.75	0.229

For categorical variables, 0 indicates ‘female’ or ‘without comorbid disease’ and 1 indicates ‘male’ or ‘with comorbid disease’. The covariables included in the regression model are: gender, age, BMI, ESS score, smoking history, hypertension, CAD, COPD, GERD, chronic pharyngitis, SL, MAI, AHI, nadir SpO_2_, mean SpO_2_, ODI, and SIT_90_. The comorbid diseases were not included as covariables when they were treated as dependent variables. OSA: obstructive sleep apnea. T2DM: type 2 diabetes mellitus. CAD: coronary artery disease. COPD: chronic obstructive pulmonary disease. GERD: gastroesophageal reflux disease.

* p-values indicate statistical significance.

## DISCUSSION

This study showed that 22% of newly diagnosed OSA patients in China were current/former smokers. Compared to non-smokers, the proportion of males in current/former smoking OSA patients was higher, with a slightly higher mean age and number of elderly patients. In addition, the ESS scores of the current/former smoking OSA patients was higher, with a longer SL and lower nocturnal mean oxygen saturation, but no difference in AHI compared with the non-smoking OSA patients. Smoking history was associated with COPD, hypertension, GERD and chronic pharyngitis, which are listed in descending order according to their strength of association.

Smoking negatively affects some physiological parameters during sleep, including sleep architecture and nocturnal oxygenation. Previous studies by Jaehne et al.^21^ and Zhou et al.^22^ have shown that smoking prolongs SL. Another study by Casasola et al.^8^ suggested that smoking reduces the oxygen saturation of healthy people at night. In OSA patients, smokers have been shown to have a lower average oxygen saturation at night^5^ than non-smokers. Our results were consistent with the findings of the above studies. Like the studies of Hsu et al.^7^ and Casasola et al.^8^, this study did not show an association between smoking history and the severity of OSA as represented by AHI; however, the ESS score of current/former smokers was slightly higher than in non-smokers. Daytime sleepiness is related to a variety of factors. For example, it is related to the role and dependence of nicotine^23^. In OSA patients, daytime sleepiness may be related to AHI, sleep fragmentation, and nocturnal hypoxia^24^. Our previous study has shown that in addition to AHI, changes of sleep architecture, SL, and nocturnal mean oxygen saturation are independent predictors of excessive daytime sleepiness^16^. Therefore, we speculate that the higher ESS score of smoking OSA patients may be related to the combined effects of nicotine, changes in sleep architecture, and nocturnal hypoxia.

The relationship between OSA and cardiovascular diseases has been confirmed by numerous studies. Hypertension is the most common cardiovascular disease and the most common comorbidity in OSA patients. The incidence of hypertension in OSA patients is 35–80%^25^, which was also confirmed in our study. In addition, smoking is one of the important risk factors for hypertension by inducing mitochondrial oxidative stress and endothelial damage^26^. Our study suggests a significant increase in the risk of hypertension associated with current/former smoking OSA patients. Contrary to our findings, Bielicki et al.5 showed that the proportion of non-smoking OSA patients with hypertension was higher than that of the smokers. This variation may be related to the discrepancy in the study population. The smoking OSA patients from Bielicki et al. were younger than the OSA patients of the current/former smokers in our study, and the incidence of hypertension increases with age^27^.

The relationship between OSA and COPD is complicated. On the one hand, both are common diseases, and thus, the probability of coexistence is not low. On the other hand, different clinical phenotypes of COPD have different effects on OSA. For example, a predominant emphysema phenotype with significant emphysema, a large lung volume, and a lower BMI, has reduced probability of OSA. A higher lung volume causes the mediastinal tissues to be pulled caudally, leading to upper airway dilation, and the increase of functional residual capacity can also buffer the fluctuation of blood gas and reduce the possible high loop gain in some patients^28^. While a predominant chronic bronchitis phenotype associated with peripheral edema and a higher BMI is more prone to OSA^3^. Smoking is the most important risk factor for COPD, and a considerable number of COPD patients continue to have airway inflammation after smoking cessation, with their lung function continuing to deteriorate and the disease continuing to progress^29^. OSA patients with a smoking history also have increased incidence of COPD. Our results confirmed the above point of view. Compared with other comorbidities associated with OSA, smoking had the strongest association with COPD.

In this study, in addition to COPD and hypertension, GERD was the third comorbidity associated with OSA that was closely related to smoking. Recurrent obstructive apnea causes increased intrathoracic pressure, which easily leads to acid reflux and promotes the occurrence of GERD^30^. A meta-analysis by Wu et al.^4^ has shown that the risk of GERD in OSA patients is 1.75-fold higher than in non-OSA patients. In addition, smoking is also an established risk factor for GERD12. A recent study of 1518 tested subjects^31^ has shown that smokers are 1.52 times more likely to develop GERD than non-smokers. However, it is unknown whether smoking cessation reduces the risk of GERD^12^. Another study by Ness-Jensen et al.^32^ has shown that smoking cessation only reduces severe reflux symptoms in individuals with a normal BMI. Therefore, the effect of smoking cessation on GERD in OSA patients with a relatively high BMI remains to be investigated.

Both vibration trauma during apnea events/snoring and CIH in OSA patients can lead to inflammation and edema of the upper airway^28,33^. Moreover, inflammation may induce or aggravate OSA by changes in local tissues and physiological reflexes^28,34^. Therefore, upper airway inflammation is very common in OSA patients^35^ and it seems to be both a cause and consequence of OSA^28^. This was also confirmed in our current study. The mucosa of the upper respiratory tract is easily affected by smoke. Long-term smoking causes edema, hyperplasia, hypertrophy, and ciliary dysfunction of mucosa of the upper airway^7,36^, leading to chronic inflammation of the upper airway, which in turn changes the biomechanical properties of the upper airway^37^, increasing its instability. In addition, nicotine damages the neuromuscular protective reflex^7^. The above factors are important in the pathogenesis of OSA. Therefore, we speculate that smoking may play an important role in promoting inflammation within the upper airway and the formation of a vicious cycle of OSA.

### Limitations

In this study, the medical record system of our hospital did not include the smoking history of the patients in detail, i.e. the total cigarette pack-year history. Instead, it only recorded the cigarette smoking status: ever-smoking or never-smoking. Since the detailed cigarette pack-year history was not obtained at the time of patients’ visit, we combined current and former smokers into one group. Therefore, this study could only show the results of patients with a history of smoking, and subgroup analysis of smoking status was not available. Given the retrospective nature of the study, this is inevitable and we will address this limitation in future well-designed prospective studies.

## CONCLUSIONS

This study showed that more than one in five newly diagnosed OSA patients in China have a history of smoking. The proportion of men with OSA who had a history of smoking was higher than that of nonsmokers. Compared with the non-smokers, OSA patients with a history of smoking had a longer SL and a lower nocturnal mean oxygen saturation, but no difference in AHI. OSA patients with a history of smoking were more likely to have hypertension, COPD, GERD, and chronic pharyngitis. This is the first study to explore the relationship between smoking history and OSA comorbidities in Chinese patients. The combined detrimental effects of smoking and OSA on comorbidities and patient outcomes need to be further validated by future studies.
